# Designing a Mission statement Mobile app for palliative care: an innovation project utilizing design-thinking methodology

**DOI:** 10.1186/s12904-020-00659-1

**Published:** 2020-10-06

**Authors:** Rakhshan Kamran, Arianna Dal Cin

**Affiliations:** 1 Michael G. DeGroote School of Medicine, McMaster University, MDCL 3114, 1280 Main Street West, Hamilton, ON L8S 4 K1 Canada; 2 Division of Plastic Surgery, McMaster University, 1280 Main Street West, Hamilton, ON L8S 4 K1 Canada

**Keywords:** Palliative care, Advance care planning, Design-thinking, Technology, App

## Abstract

**Background:**

Eliciting individual values and preferences of patients is essential to delivering high quality palliative care and ensuring patient-centered advance care planning. Despite advance care planning conserving healthcare costs by up to 36%, reducing psychological distress of patients and caregivers, and ensuring palliative care delivery in line with patient wishes, less than 33% of adults engage in it. We aimed to develop a mobile application intervention to address the challenges related to advance care planning and improve the delivery of palliative care.

**Methods:**

Design-thinking methodology was used to develop a mobile application, in response to issues prominently identified in current palliative care literature.

**Results:**

Issues surrounding communication of patient values from both the patient and provider side is identified as a main issue in palliative care. We designed a mobile application intervention prototype to address this.

**Conclusions:**

Our “Mission Statement” mobile application will allow patients to create a mission statement identifying what they want their care team to know about them, as well as space to identify important values and preferences. Patients will be able to evolve their mission statement and values and preferences over the course of their palliative care journey through the application. Design-thinking methodology is an effective tool to drive healthcare innovation and bridge the gap between research findings and implementation.

## Background

Palliative care is a dynamic, interdisciplinary, and highly personalized process [1]. It aims to not hasten or delay death, rather, focus on pain and symptom management, improve quality of life, and emotional and spiritual care [1]. Palliative care also involves advance care planning, which includes reflection on one’s values and beliefs and then communicating them with healthcare providers and family members [2–4]. The benefits of advance care planning are well documented. Advance care planning allows patients to receive care which is in line with their values and preferences [5, 6], allows caregivers and family members of patients to have reduced psychological distress over being a surrogate decision maker and interpreting unknown or ambiguous patient wishes [7, 8], and conserves healthcare costs by up to 36% [9, 10]. Many guidelines also report on the importance of honest and compassionate communication in end-of-life care being essential for patient-centeredness [11–13].

Despite these benefits, less than 33% of adults have engaged in advance care planning conversations or completed advance directives due to conversations around death and dying being difficult and uncomfortable [14]. Healthcare providers also face difficulty in having conversations around death and dying with patients, and in eliciting the values and preferences of patients [15]. Further, while patients report on communication as important for high quality care [16, 17], they also report dissatisfaction with physician communication [18]. In palliative care, where communication between patients and physicians is delicate, complex and particularly important [19], poor communication in these settings result in anxiety, depression and dissatisfaction with care amongst patients and their families [20–23].

New approaches for conversations in palliative care and with advance care planning include the use of board games in community settings which use gamification to decrease stigma around conversations around death [24, 25], and using patient-reported outcome measures (PROMs) as a communication tool [26, 27]. However, the feasibility of implementing board games in a clinical setting is currently unknown [8, 9]. Further, there is no current valid and reliable PROM found acceptable for use in palliative care [24–28].

Our team is responding to the need of improving patient communication in palliative care and advance care planning through creating a mobile application to track patient values and preferences. We are designing a mobile application which will allow patients to create a patient “mission statement”, and provide an overview of their goals, values, and wishes; which will be personalized by each patient.

A mobile application measuring patient values and creating a patient mission statement will also aid in the provision of care [29]. Through having healthcare providers conveniently, at-a-glance view the concepts most important to patients, there will be less ambiguity around patient values and preferences, which is a current problem in palliative care and advance care planning [7, 8, 30, 31]. Through having a mobile application, patients will be able to input concepts important to them and healthcare providers will be able to use it as a tool for conversation and direct care to make patients feel less burdened to repeat their information over again as well.

A mobile application tracking quality of life and values, along with a patient mission statement will also address the problem of caregiver psychological distress when making decisions around ambiguous patient values [7, 8]. Allowing patients to be in control of having their values and preferences inputted into the mobile application and displayed to caregivers and the care team will reduce ambiguity around patient values.

The aim of this paper is to provide an overview of the design-thinking methods used to drive the first phase of development of this innovation. Results from published literature documenting issues in palliative care were leveraged to create our mission statement mobile application. Subsequent phases of development will include iterative prototyping and testing the application with stakeholders, using design-thinking methodology. Our goal is for clinicians to learn about design-thinking methodology so they can lead their own design-thinking interventions to leverage insights from literature, improving care delivery and patient outcomes.

## Methods

Our team is using design-thinking methodology [32]. Design-thinking is human-centered methodology for creative problem solving [32]. It is outlined as prioritizing and using user empathy to guide solutions [32]. Collaboration between multidisciplinary teams to provide multiple perspectives on an issue, using ideation and applying qualitative research to generate potential solutions, iteratively testing solutions for the purpose of refining the solution, and creating and testing a prototype encompass the phases of design-thinking [32]. The cyclical process of continuously ideating, prototyping and testing solutions make it unique from linear or top-down approaches to designing health interventions [33]. Design thinking is widely used in the business and engineering sectors, with the design-thinking firm IDEO in partnership with Stanford University (now called the d.school and one of the most highly sought academic programs at Stanford) being the world’s largest design-thinking groups; designing products for Apple, GE, and other major companies [34, 35]. A recent systematic review identified twenty-four design-thinking interventions used in healthcare which all demonstrated increased satisfaction, usability and effectiveness compared to traditional interventions [33]. Asch and colleagues also report on design-thinking methodology as integral to creating high-impact innovation in healthcare. Asch calls on clinicians to employ design-thinking to create forward-thinking solutions to system issues [36].

Our team worked with the Health Leadership Academy at McMaster University on approaching the design of our intervention, using design-thinking methodology. Below we provide an overview of design-thinking methods.

The first phase of design-thinking methodology is to identify a problem [32]. Problem identification occurs through leveraging insights from stakeholders on their perspective, and synthesizing data to identify the most prominent issue [32]. Once a problem has been identified, a preliminary prototype is created as a solution, which is then refined and tested. Refining and testing occurs through the POEMS framework and qualitative analysis.

Observation of user interactions with a prototype is analyzed through the Peoples, Objects, Environment, Messages and Services (POEMS) framework [37]. The POEMS framework is used in the field of design-thinking to analyze observational data [37]. In summary, a structured sheet consisting of the headings “Peoples”, “Objects”, “Environment”, “Messages” and “Services” are used to organize observational data of users interacting with a protype, such as a mobile app. The “users” include all stakeholders. Data is then synthesized and combined to identify common themes regarding user interactions and the context of themes. This data is then analyzed through a bottom-up process to identify the implications of common themes and their application to refine the design of a prototype [37]. Through the process of contextual inquiry [38, 39], data results are displayed visually for the purpose of the research team to engage in peer debriefing and review the concepts as a group. Application of final themes are then applied to the prototype design. This process occurs iteratively and through each stage of development of the proposed intervention in order to refine it based on user experience.

Qualitative analysis also occurs through interviewing relevant stakeholders [32]. Semi-structured interviews are conducted to gather stakeholder perspective on a prototype [32]. Additional probing questions are asked in design-thinking methodology to elicit the way stakeholders feel and think about a prototype, in addition to their thoughts on interacting with the prototype [32]. Qualitative interviews are then transcribed verbatim and analyzed to identify common themes from across stakeholders. This data is also analyzed through a bottom-up process to identify the implication of themes to refine the prototype.

Through synthesizing observational and qualitative data, a prototype is iteratively refined in a cyclical nature.

### Current status of project

This paper highlights the first phase of our project. Previously published literature, which engaged stakeholders in palliative care, was used to identify prominent issues in palliative care to design a preliminary mobile application. Current literature in palliative care identifies a prominent theme of issues with communication of patient values from both the patient and provider side [15, 30, 31]. We used this information to develop a mobile application. Details on refining and testing the prototype mobile application is the focus of the next phase of our study.

The planned intervention is our “Mission Statement” mobile application. The mobile application will allow patients to create a mission statement early in the palliative care process based upon their goals and values. The mission statement will be visible for patient family members and members of the healthcare team through patients sharing their mission statement with healthcare providers and family, who will also have a version of the mobile application. The mission statement will be adaptable as the patient progresses through their treatment course in order to clearly identify changing patient wishes and values along the palliative care course. This intervention will create empowerment and agency in palliative care, driven and led by what the patient’s values and preferences are.

Our aims for the intervention are to reduce ambiguity in palliative care around patient goals and values and having the patient voice at the forefront rather than lost in translation.

### Components of application

Currently, the mobile application is proposed to have three main components. Firstly, an open space for the patient to write a mission statement embodying the main message they would like others to know about them that may help caregivers and healthcare providers with their provision of care. Secondly, a page below for patients to respond to questions in an open-ended manner which have been identified as important for palliative care patients (e.g., what music would you want to listen to on your last day alive) [24–28]. Thirdly, a final section which will allow patients to group important values to them. Patients will be able to click and drag values such as “being in as little pain as possible”, “having my whole family with me”, and “being able to use my hands” into categories: “very important”, “a little important”, “not very important”. Each component of the app will have the flexibility for patients to write their own preferred information to maintain the app being as patient-centered and patient-focused as possible. Visuals on the final version of the application will be provided once the prototyping phase is complete.

Our proposed app will be as simple to use as possible, will act as a way to display patient values and preferences, and as a communication tool between patients, caregivers, and physicians. Stakeholder input will also be used at every step of the way in refining this prototype, following design-thinking methodology.

### Future direction of project

We will be using design-thinking methodology to iteratively refine our mobile application to include patient and stakeholder input at every step of the way. We will begin by creating a low-fidelity coded prototype and collect qualitative and observational data from patient and provider groups. Using synthesized feedback, our team will refine the mobile application. Large-scale user testing will occur with our final prototype. We will administer our mobile application and pilot test it at hospitals and hospices in a large urban center, using the POEMS framework to collect and analyze observational data. Qualitative feedback will also be collected from healthcare providers and patients through interviews which will measure their experience of how the app has impacted process of care and communication in palliative care.

Design-thinking uses qualitative research methods, including ethnographic observations, in order to gain rich information about the users for which the interventions are tailored to [40, 41]. Design-thinking has shown in some cases to have a bias towards action (i.e., implementing a final product without adequate user input) [40]. Proposed methods of protecting against this source of bias includes testing minimally viable products early in the design phase in order to learn from users and stakeholders how to improve them [40]. Through creation of our intervention, we will iteratively test our mobile application in line with its stages of development to gain user and stakeholder input at every stage. Through including users iteratively, we are also minimizing social desirability bias [42].

## Discussion

Our proposed idea introduces a novel mobile application intervention to improve the quality of palliative care delivered and to improve the patient, provider, and caregiver experience around advance care planning. We will continue to use user-centered design-thinking methodology to create a mobile application measuring patient values and preferences, and creating a mission statement for use with patients undergoing palliative care. Through receiving input from patients, caregivers, and healthcare providers, our team will tailor our intervention, as outlined in design-thinking methodology [32], to ensure our intervention is created using patient and provider input. The results from our study will create an innovative intervention which will enhance patient-provider communication in palliative care and empower patients in this delicate area of care [43, 44].

A systematic review conducted by Slort and colleagues identified barriers and facilitators to end of life communication from both the patient and clinician level [15]. At the patient level, barriers to end of life communication identified were ambivalent patient attitudes about prognosis, not talking about their problems and needs, and changing ideas and preferences as their disease progresses [15]. At the clinician level, lack of time, difficulty in initiating conversation on an issue, personal obstacles (e.g., dealing with patient denial) and not taking initiative to contact patients spontaneously were barriers to communication [15]. Facilitators at the clinician level included taking initiative to talk about end of life issues, shared decision making, being open, and learning about patient preferences [15]. Slort and colleagues recommended that clinicians need to continuously re-appraise their patient’s needs and preferences and have a high level of communication skills to discuss emotional and spiritual end of life issues with patients [15].

A recent review conducted by Noordman and colleagues identified current strategies and tools in use by healthcare providers for communication in palliative care [30]. Face-to-face communication strategies include: the teach-back method where patients repeat back information communicated to them, jargon-free communication, and adopting a slow rate of speech [30]. Written and online strategies include supplemental graphs and illustrations for conversation, and using short sentences and paragraphs [30]. Tools (patient decision aids and question prompt lists) were also identified in one study to facilitate patient-provider communication [30]. A structured list of questions for healthcare providers to ask patients was found to empower patients to discuss prognosis and end of life issues, and reduce decision burden [30].

Further, a review by McCaffery identified that most patient decision aids and question prompt lists are not designed and tested with patients, rendering them inadequate [31]. Noordman and colleagues specifically recommend using emerging technologies and media with user-centered design for future research and to practice design technology-mediated innovations for patients in palliative care [31].

Our proposed initiative addresses the aforementioned barriers to end of life communication from the patient and clinician perspective such as lack of time, difficulty initiating conversation, and the need to constantly re-appraise patient needs and preferences. Our mobile application is also responding to the need of a patient decision aid in palliative care being created using user-centered design and leveraging emerging technology, and *applying* the important insights gained from previous research.

## Conclusion

Through using design-thinking methodology, our mission statement mobile application is an innovation project, designed within healthcare; rather than copy and pasting a solution from another sector and applying it to healthcare. Identifying issues with communication in palliative care, covered extensively in current palliative care literature, led us to create our Mission Statement mobile application intervention. Leveraging user insights from literature findings to design creative solutions to complex problems in healthcare for the purpose of improving care delivery can be done effectively through design-thinking methodology. Our intervention highlights an example of applying literature findings and creating a solution to a problem through looking within healthcare for a solution rather than insourcing innovation from other fields. Due to clinicians working directly with patients, they are uniquely positioned to develop innovative interventions through understanding patient experience. Design-thinking methodology is an innovation principle which clinicians can apply in healthcare to create interventions which improve patient experience and outcomes.

## Data Availability

Not applicable (not reporting individual data).

## Abbreviations

PROM: Patient Reported Outcome Measure
POEMS: People, Objects, Environment, Messages and Services

## References

[1] Health Canada (2018). Framework on Palliative Care in Canada.

[2] Committee on Approaching Death: Addressing Key End of Life Issues, Institute of Medicine (2015). Dying in America: Improving Quality and Honoring Individual Preferences Near the End of Life.

[3] Sudore RL, Schickedanz AD, Landefeld CS, Williams BA, Lindquist K, Pantilat SZ (2008). Engagement in multiple steps of the advance care planning process: a descriptive study of diverse older adults. J Am Geriatr Soc.

[4] Fried TR, Bullock K, Iannone L, O’Leary JR (2009). Understanding advance care planning as a process of health behavior change. J Am Geriatr Soc.

[5] Silveira MJ, Kim SYH, Langa KM (2010). Advance directives and outcomes of surrogate decision making before death. N Engl J Med.

[6] Detering KM, Hancock AD, Reade MC, Silvester W (2010). The impact of advance care planning on end of life care in elderly patients: randomised controlled trial. BMJ..

[7] Kramer BJ, Boelk AZ, Auer C (2006). Family conflict at the end of life: lessons learned in a model program for vulnerable older adults. J Palliat Med.

[8] Detering K, Sutton E, Fraser S, Wallis K, Silvester W, Mawren D (2015). Feasibility and acceptability of advance care planning in elderly Italian and Greek speaking patients as compared to English-speaking patients: an Australian cross-sectional study. BMJ Open.

[9] Nicholas LH, Langa KM, Iwashyna TJ, Weir DR (2011). Regional variation in the association between advance directives and end-of-life Medicare expenditures. JAMA..

[10] Zhang B, Wright AA, Huskamp HA, Nilsson ME, Maciejewski ML, Earle CC (2009). Health care costs in the last week of life: associations with end-of-life conversations. Arch Intern Med.

[11] Davidson JE, Powers K, Hedayat KM, Tieszen M, Kon AA, Shepard E (2007). Clinical practice guidelines for support of the family in the patient-centered intensive care unit: American College of Critical Care Medicine Task Force 2004-2005. Crit Care Med.

[12] Dorman T, Angood PB, Angus DC, Clemmer TP, Cohen NH, Durbin CG (2004). Guidelines for critical care medicine training and continuing medical education. Crit Care Med.

[13] Nici L, ZuWallack R (2012). American Thoracic Society Subcommittee on integrated Care of the COPD patient. An official American Thoracic Society workshop report: the integrated Care of the COPD patient. Proc Am Thorac Soc.

[14] Rao JK, Anderson LA, Lin F-C, Laux JP (2014). Completion of advance directives among U.S. consumers. Am J Prev Med.

[15] Slort W, Schweitzer B, Blankenstein A, Abarshi E, Riphagen I, Echteld M (2011). Perceived barriers and facilitators for general practitioner–patient communication in palliative care: a systematic review. Palliat Med.

[16] Abbott KH, Sago JG, Breen CM, Abernethy AP, Tulsky JA (2001). Families looking back: one year after discussion of withdrawal or withholding of life-sustaining support. Crit Care Med.

[17] Curtis JR, Wenrich MD, Carline JD, Shannon SE, Ambrozy DM, Ramsey PG (2002). Patients’ perspectives on physician skill in end-of-life care: differences between patients with COPD, cancer, and AIDS. Chest..

[18] Azoulay E, Chevret S, Leleu G, Pochard F, Barboteu M, Adrie C (2000). Half the families of intensive care unit patients experience inadequate communication with physicians. Crit Care Med.

[19] Heyland DK, Groll D, Rocker G, Dodek P, Gafni A, Tranmer J (2005). End-of-life care in acute care hospitals in Canada: a quality finish?. J Palliat Care.

[20] Curtis JR, Patrick DL, Caldwell E, Greenlee H, Collier AC (1999). The quality of patient-doctor communication about end-of-life care: a study of patients with advanced AIDS and their primary care clinicians. AIDS..

[21] Weiner JS, Roth J (2006). Avoiding iatrogenic harm to patient and family while discussing goals of care near the end of life. J Palliat Med.

[22] Heyland DK, Rocker GM, O’Callaghan CJ, Dodek PM, Cook DJ (2003). Dying in the ICU: perspectives of family members. Chest..

[23] Heyland DK, Allan DE, Rocker G, Dodek P, Pichora D, Gafni A (2009). Discussing prognosis with patients and their families near the end of life: impact on satisfaction with end-of-life care. Open Med.

[24] Van Scoy LJ, Watson-Martin E, Bohr TA, Levi BH, Green MJ (2018). End-of-life conversation game increases confidence for having end-of-life conversations for chaplains-in-training. Am J Hosp Palliat Care.

[25] Van Scoy LJ, Reading JM, Hopkins M, Smith B, Dillon J, Green MJ (2017). Community game day: using an end-of-life conversation game to encourage advance care planning. J Pain Symptom Manag.

[26] Antunes B, Harding R, Higginson IJ (2014). Implementing patient-reported outcome measures in palliative care clinical practice: a systematic review of facilitators and barriers. Palliat Med.

[27] Bausewein C, Simon ST, Benalia H, Downing J, Mwangi-Powell FN, Daveson BA (2011). Implementing patient reported outcome measures (PROMs) in palliative care - users’ cry for help. Health Qual Life Outcomes.

[28] Simon ST, Higginson IJ, Harding R, Daveson BA, Gysels M, Deliens L (2012). Enhancing patient-reported outcome measurement in research and practice of palliative and end-of-life care. Support Care Cancer.

[29] Mamlin BW, Tierney WM (2016). The promise of information and communication Technology in Healthcare: extracting value from the chaos. Am J Med Sci.

[30] Noordman J, van Vliet L, Kaunang M, van den Muijsenbergh M, Boland G, van Dulmen S (2019). Towards appropriate information provision for and decision-making with patients with limited health literacy in hospital-based palliative care in Western countries: a scoping review into available communication strategies and tools for healthcare providers. BMC Palliative Care.

[31] McCaffery KJ, Holmes-Rovner M, Smith SK, Rovner D, Nutbeam D, Clayman ML (2013). Addressing health literacy in patient decision aids. BMC Med Inform Decis Mak.

[32] Brown T (2008). Design thinking. Harv Bus Rev.

[33] Altman M, Huang TT, Breland JY (2018). Design Thinking in Health Care. Prev Chronic Dis.

[34] Perlroth N (2013). Solving Problems for Real World, Using Design. The New York Times.

[35] Johansson-Sköldberg U, Woodilla J, Çetinkaya M (2013). Design thinking: past, present and possible futures. Creat Innov Manag.

[36] Asch DA, Terwiesch C, Mahoney KB, Rosin R (2014). Insourcing health care innovation. N Engl J Med.

[37] Kumar V. POEMS 101 design methods: a structural approach for driving innovation in your organization. Hoboken: Wiley; 2012.

[38] Beyer H, Holtzblatt K (1999). Contextual design. Interactions.

[39] Martin B, Hanington B (2012). Universal methods of design: 100 ways to research complex problems, develop innovative ideas and design effective solutions.

[40] Ries E. The lean startup: how today’s entrepreneurs use continuous innovation to create radically successful businesses. New York: Random House LLC; 2011.

[41] Raghu A, Praveen D, Peiris D, Tarassenko L, Clifford G (2015). Engineering a mobile health tool for resource-poor settings to assess and manage cardiovascular disease risk: SMARThealth study. BMC Med Inform Decis Mak.

[42] Hecht ML (1978). The conceptualization and measurement of interpersonal communication satisfaction. Hum Commun Res.

[43] Scott AM, Caughlin JP (2014). Enacted goal attention in family conversations about end-of-life health decisions. Commun Monogr.

[44] Clayton JM, Butow PN, Waters A, Laidsaar-Powell RC, O’Brien A, Boyle F (2013). Evaluation of a novel individualised communication-skills training intervention to improve doctors’ confidence and skills in end-of-life communication. Palliat Med.

